# Small Bed-Chambers

**Published:** 1882-04

**Authors:** 


### SMALL BED-CHAMBERS.

There is reason to believe that more cases of dangerous and fatal disease are gradually engendered annually by the habit of sleeping in small, unventilated rooms, than have occurred from a cholera atmosphere during any year since it made its appearance in this country. Very many persons sleep in eight by ten rooms, that is, in rooms the length and breadth of which multiplied together, and this multiplied again by ten for the height of the chamber, would make just eight hundred cubic feet, while the cubic space for each bed, according to the English apportionment for hospitals, is twenty-one hundred feet. But more, in order "to give the air of a room the highest degree of freshness," the French hospitals contract for a complete renewal of the air of a room every hour, while the English assert that double the amount, or over four thousand feet an hour, is required. Four thousand feet of air every hour! and yet there are multitudes in the city of New-York who sleep with closed doors and windows in rooms which do not contain a thousand cubic feet of space, and that thousand feet is to last all night, at least eight hours, except such scanty supplies as may be obtained of any fresh air that may insinuate itself through little crevices by door or window, not an eighth of an inch in thickness. But when it is known that in many cases a man and wife and infant sleep habitually in thousand-feet rooms, it is no marvel that multitudes perish prematurely in cities; no wonder that infant children wilt away like flowers without water, and that five thousand of them are to die in the city of New-York alone during the hundred days which shall include the fifteenth of July, eighteen hundred and ! Another fact is suggestive, that among the fifty thousand persons who sleep nightly in the lodging-houses of London, expressly arranged on the improved principles of space and ventilation already referred to, it has been proven that not one single case of fever has been engendered in two years! Let every intelligent reader improve the teachings of this article without an hour's delay.

### EACH THE BEST.

A GENTLEMAN called for medical advice the other day, whose reputation as the manufacturer of a certain article of hardware would sell any thing with his name upon it. On being asked if the quality of the article depended on any secret mode of preparation, or any peculiarity of handwork, or any patent protection, he replied: "Nothing of the sort; the article is not patented, nor is there any secret in my establishment. On the contrary, while my process is peculiar, it has at the same time been made public, and I have debated the propriety of my course, and defended it against my friends and rivals in the same business. I took my stand at the age of twenty years, and have resolutely adhered to it for nearly a third of a century through every phase of the market; there is no year, of all that time, in which we failed to make money; although, at times, we may have been compelled to sell at actual cost. But now, for many years, I have had the preference in the markets of the world; seeing this, my name was pirated by thirty manufacturers in Manchester, at one and the same time, and in every single case, the English courts decided in my favor, and compelled the rectification of the wrong. My plan was simply this; I allowed my name to be stamped, only on the very best article my establishment could turn out. I had a finished expert in each particular branch and stage of the manufacture, to inspect the article; he had nothing else to do, and it was not 'passed,' until each one had declared that it was without a flaw. At first, multitudes were thrown aside, but by rigidly requiring each department to strive to make each successive article the best of any that preceded it, we now habitually make '*each the best*,' and this is the answer to your question." So he will live longer, who will strive to make each day the best, in industrious exercise and a comprehensive temperance; who, if he work too little to-day for a good night's rest, will strive to work more to-morrow; if he eats too much to-day for subsequent comfort, will retrench to-morrow, or so modify his eating in quantity or quality, that there will be no unpleasant reminder after any meal, that an excess has been committed; this apparently little thing has engaged the attention, and waked up the resolves, and baffled the efforts of the greatest minds in all ages; showing that a brute appetite wars daily with a nature divine, as, in handicraft, cupidity tempts integrity and generally overcomes it; hence so few men, in any branch of business, legitimately succeed in the long run. Let every nurse, then, endeavor to do better with each succeeding invalid; let every physician endeavor to give more thought and attention to each new patient, and let us all, in all things, strive patiently, resolutely, persistently, to make each day "the best" of any that preceded it, and thus do more than could possibly be done in any other way, to prevent our lives being a failure; a calamity than which no greater could befall any man.

